# Effect of Annealing and Thickness of Co_40_Fe_40_Yb_20_ Thin Films on Various Physical Properties on a Glass Substrate

**DOI:** 10.3390/ma15238509

**Published:** 2022-11-29

**Authors:** Wen-Jen Liu, Yung-Huang Chang, Chia-Chin Chiang, Chi-Lon Fern, Yuan-Tsung Chen, Ying-Hsuan Chen, Hao-Wen Liao, Te-Ho Wu, Shih-Hung Lin, Ko-Wei Lin, Po-Wei Chi

**Affiliations:** 1 Department of Materials Science and Engineering, I-Shou University, Kaohsiung 84001, Taiwan; 2 Bachelor Program in Interdisciplinary Studies, National Yunlin University of Science and Technology, 123 University Road, Section 3, Douliou 64002, Taiwan; 3 Department of Mechanical Engineering, National Kaohsiung University of Science and Technology, 415 Chien Kung Road, Kaohsiung 80778, Taiwan; 4 Department of Materials Science and Engineering, National Chung Hsing University, Taichung 40227, Taiwan; 5 Graduate School of Materials Science, National Yunlin University of Science and Technology, 123 University Road, Section 3, Douliou 64002, Taiwan; 6 Department of Electronic Engineering, National Yunlin University of Science and Technology, 123 University Road, Section 3, Douliou 64002, Taiwan; 7 Institute of Physics, Academia Sinica, Nankang, Taipei 11529, Taiwan

**Keywords:** X-ray diffraction (XRD), adhesion, transmittance, electrical properties, nanomechanical property, low-frequency alternating current magnetic susceptibility (χ_ac_)

## Abstract

The aim of this work is to investigate the effect of annealing and thickness on various physical properties in Co_40_Fe_40_Yb_20_ thin films. X-ray diffraction (XRD) was used to determine the amorphous structure of Co_40_Fe_40_Yb_20_ films. The maximum surface energy of 40 nm thin films at 300 °C is 34.54 mJ/mm^2^. The transmittance and resistivity decreased significantly as annealing temperatures and thickness increased. At all conditions, the 10 nm film had the highest hardness. The average hardness decreased as thickness increased, as predicted by the Hall–Petch effect. The highest low-frequency alternative-current magnetic susceptibility (χ_ac_) value was discovered when the film was annealed at 200 °C with 50 nm, and the optimal resonance frequency (ƒ_res_) was in the low frequency range, indicating that the film has good applicability in the low frequency range. At annealed 200 °C and 50 nm, the maximum saturation magnetization (Ms) was discovered. Thermal disturbance caused the Ms to decrease when the temperature was raised to 300 °C. The optimum process conditions determined in this study are 200 °C and 50 nm, with the highest Ms, χ_ac_, strong adhesion, and low resistivity, which are suitable for magnetic applications, based on magnetic properties and surface energy.

## 1. Introduction

CoFe alloys are commonly used in a variety of magnetic devices, such as sensors, actuators, read heads, and magnetic random-access memory (MRAM) [1,2,3]. Most non-crystal glass substrates used for perpendicular magnetic anisotropy (PMA) have a certain degree of rigidity and flatness [4,5]. The soft magnetic underlayer is very helpful for the writing of perpendicular magnetic recording. The role is to help the single pole writer with magnetic field line transmission. It can be written into the material with higher magnetic anisotropy constant (K_u_) values. Therefore, it has the potential to improve the thermal stability of the recording medium. The magnetic properties of a CoFe (Cobalt Iron) alloy can be tuned depending on the application by varying the Co to Fe ratio [6,7]. However, the CoFe alloy does not have low coercivity (Hc), and the annealing temperature accelerates the degeneration of magnetic anisotropy faults, making it difficult to meet the magnetic equipment used at high temperatures. One solution for increasing the thermal stability of the CoFe alloy is to add a third element. The common characteristics of rare earth magnetic materials are high saturation magnetization (Ms), Curie temperature (Tc), and magnetic anisotropy field (H_k_), particularly. In general, the addition of rare-earth (RE) elements to CoFe alloy can improve its hardness, corrosion resistance, and durability, as well as improve heat resistance and other benefits [8,9,10]. Due to their widespread use in numerous applications, including spintronic, fiber amplifier, photoluminescence, and laser, RE element-doped nanomaterials have become the center of interest. As a result, the primary research goal of this paper is to identify other elements that can be added to CoFe alloys to effectively improve their stability. Ytterbium (Yb) is an important RE element because of its unique optical and magnetic properties, as well as its incompletely filled 4f electronic states [11,12,13]. YAG (Yttrium Aluminum Garnet) nanophosphor powder doped with Yb^3+^ is an excellent laser material [14]. Moreover, a free or pinned layer of Co_40_Fe_40_B_20_ thin film is frequently utilized in magnetic tunneling junction (MTJ) structures because it has a high tunneling magnetoresistance (TMR) ratio. To test several specific features, this study predominantly formed CoFeYb films using the same Yb and B (Boron) ratio. The innovative aspects of this research include examining annealed CoFeYb thin films to determine if they would change in high temperature environments, as well as investigating the structure and magnetic properties of CoFeYb thin films as a function of thickness. The performance is more sensitive to high operating temperatures and room temperature (RT). However, a few studies on the magnetic, electrical, optical, and nanomechanical properties of CoFeYb films under as-deposited and annealed conditions have been conducted. For the convenience of readers, the abbreviations and full names of proper nouns in this article are listed in Table 1. This study also looks at the thicknesses of CoFeYb in various as-deposited and annealed conditions. Previous research has compared the magnetic and adhesive properties of CoFeYb materials to those of CoFeV and CoFeW in Table 2 [15,16,17].

## 2. Materials and Methods 

**Figure 1:**
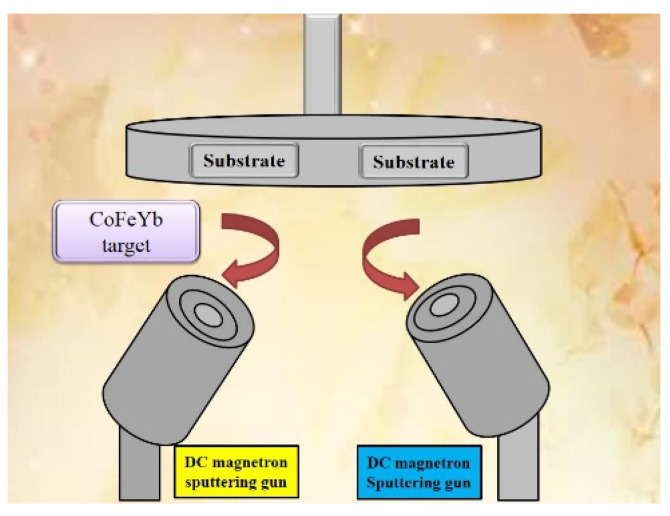
Schematic diagram of sputtering system.

CoFeYb with a thickness of 10–50 nm was sputtered onto a glass substrate at room temperature (RT) using a magnetron sputtering direct current (DC) method with a power of 50 W and the four conditions listed below: (a) as-deposited films were kept at RT, (b) annealed at 100 °C for 1 h, (c) annealed at 200 °C for 1 h, and (d) annealed at 300 °C for 1 h. A schematic of the experimental sputtering system is shown in Figure 1. Ar (Argon) operating pressure was 1.54 × 10^−3^ Torr and the chamber base pressure was 1.07 × 10^−7^ Torr. CoFeYb alloy with a target composition was 40% Co, 40% Fe, and 20% Yb. With a particular Ar gas, the pressure in the ex-situ annealed condition was 2.5 × 10^−3^ Torr. Grazing incidence X-ray diffraction (GIXRD) patterns acquired with CuKα1 (PAN analytical X’pert PRO MRD, Malvern Panalytical Ltd., Cambridge, UK) and a low angle diffraction incidence of around two degrees were used to analyze the crystal structure of CoFeYb films. A contact angle (θ) measuring tool (CAM-110, Creating Nano Technologies, Tainan City, Taiwan) was utilized to measure the contact angle using deionized (DI) water and glycerol, which was then used to determine the surface energy [18,19,20]. A spectral intelligent analyzer (Collimage, Taipei, Taiwan) was used to measure the transmittance of CoFeYb. The visible light wavelength ranged from 500 to 800 nm. The electrical properties are detected by four-point probe measurement (Sadhudesign, Hsinchu City, Taiwan). Using the MTS (mechanical testing and simulation) Nano Indenter XP (MTS, Minneapolis, MN, USA) with a Berkovich tip and the continuous stiffness measurement (CSM) method, the hardness of Co_40_Fe_40_Yb_20_ films was examined. Once the load was reduced to 10% of the maximum load, we removed the indent from the surface at the same rate. Measurement should be repeated ten times for each sample with the probe. The indentation load is multiplied by 40 stages, with each step’s indentation depth being recorded. In order to produce more precise data, six indentations from each sample were evaluated, and the standard deviations were averaged. The χ_ac_ analyzer (XacQuan, MagQu Co., Ltd., New Tapei City, Taiwan) and alternating gradient magnetometer (AGM, PMC, Westerville, OH, USA) were used to investigate the in-plane low-frequency alternate-current magnetic susceptibility (χ_ac_) and hysteresis loop of Co_40_Fe_40_Yb_20_ thin films.

## 3. Results

### 3.1. Structure 

**Figure 2:**
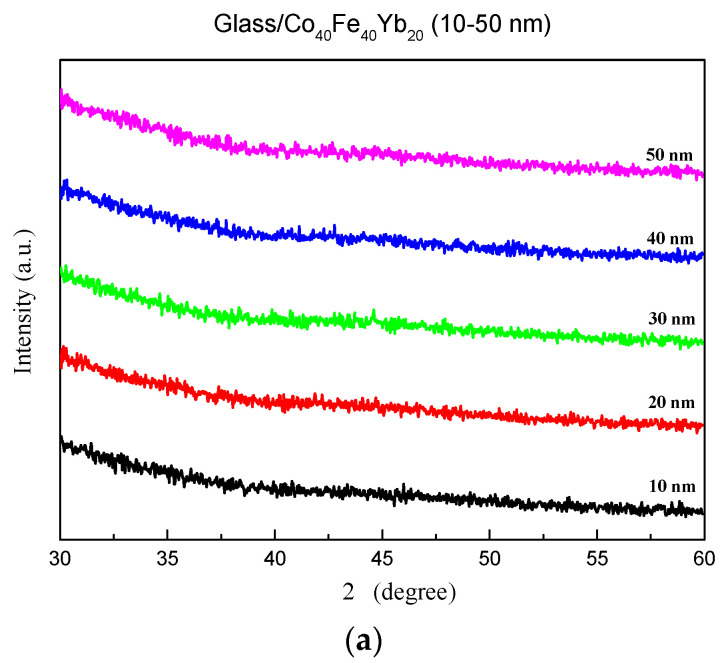

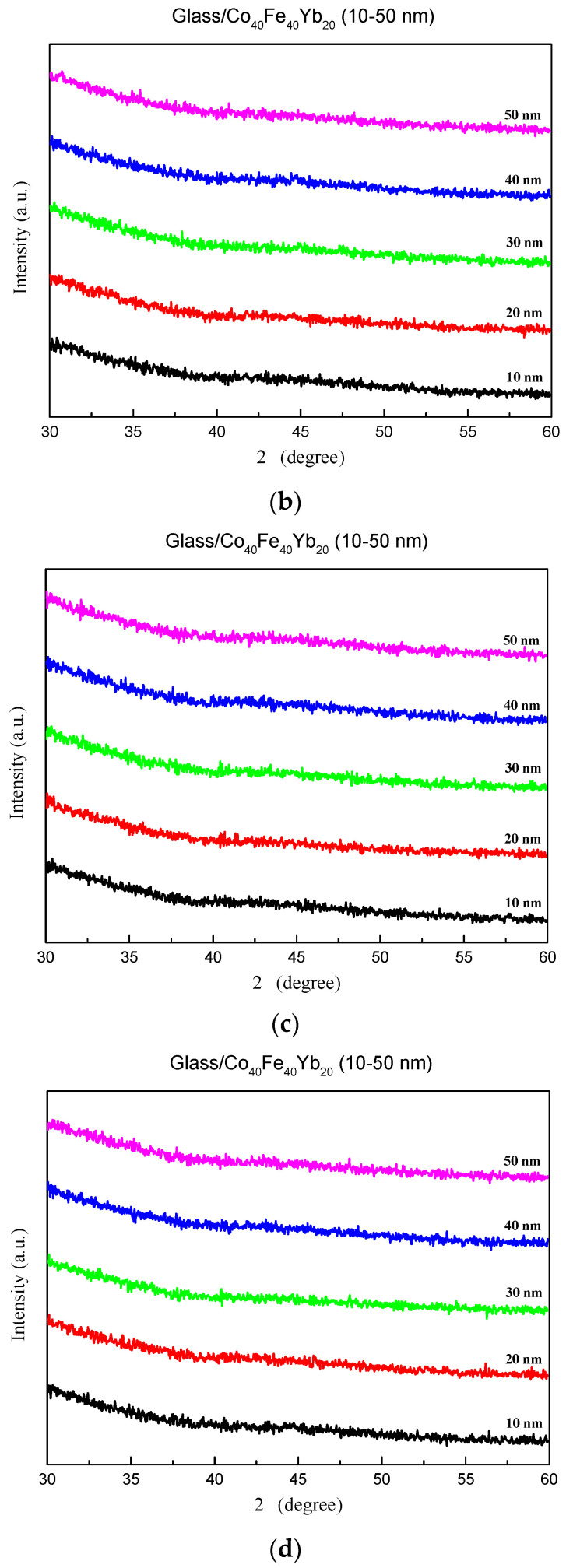
Thin films made of Co_40_Fe_40_Yb_20_ with X-ray diffraction patterns. (**a**) as-deposited, (**b**) post-annealing at 100 °C, (**c**) post-annealing at 200 °C, and (**d**) post-annealing at 300 °C.

The XRD patterns for each of the four situations are shown in Figure 2. Figure 2a–d show that the film produces no distinctive diffracted peak, indicating that it is amorphous and that the addition of Yb to CoFe alloys causes the refinement of grain size [21]. The figure also shows that the Co_40_Fe_40_Yb_20_ film sputtered on the glass substrate is in an amorphous condition and that the thermal driving force is insufficient to sustain grain growth [21,22,23,24]. The Co_40_Fe_40_Yb_20_ sputtering film is randomly arranged when the substrate is randomly arranged due to the amorphous nature of the glass substrate. When the film is deposited in thicknesses ranging from 10 nm to 50 nm, the film structure is not completely ordered, resulting in the substrate effect.

### 3.2. Analysis of Surface Energy and Adhesion

The contact angles measured under four conditions using DI water and glycerol are shown in Table 3. The Co_40_Fe_40_Yb_20_ films had contact angles that were reportedly less than 90°, and the drops were almost spherical, which led to good hydrophilicity and wettability. The contact angle can therefore be inferred to be decreasing as the higher annealed temperature increases. It is reasonable to infer that the large grains of annealed material exhibit significant gaps between adjacent grains when the grains are arranged in the material. As the gaps widen and the support between the crystal grains diminishes, the crystal grain size rises and a trend toward reduced contact angles develops. Water easily flows into gaps when it falls on a surface, producing small contact angles, strong adhesion, and high surface energies [25]. Surface energy and adhesion are critical because Co_40_Fe_40_Yb_20_ film can be used as an underlayer or buffer layer. When the surface energy is high, liquid absorption is significant and the contact angle decreases. The surface energy is calculated using Young’s equation and the contact angle [18,19,20].

**Figure 3:**
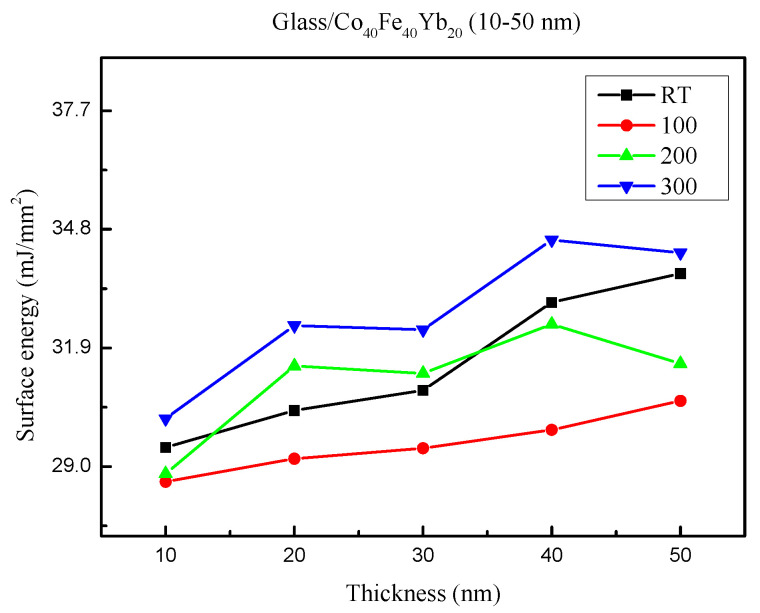
Surface energy of Co_40_Fe_40_Yb_20_ thin films.

The surface energy is depicted in Figure 3 under all conditions. The surface energy varied between 28.63 and 34.54 mJ/mm^2^. The adhesion was strongest when the films had a higher surface energy. According to the calculations, the highest surface energy of 40 nm at 300 °C was 34.54 mJ/mm^2^.

### 3.3. Examination of Optical Properties

**Figure 4:**
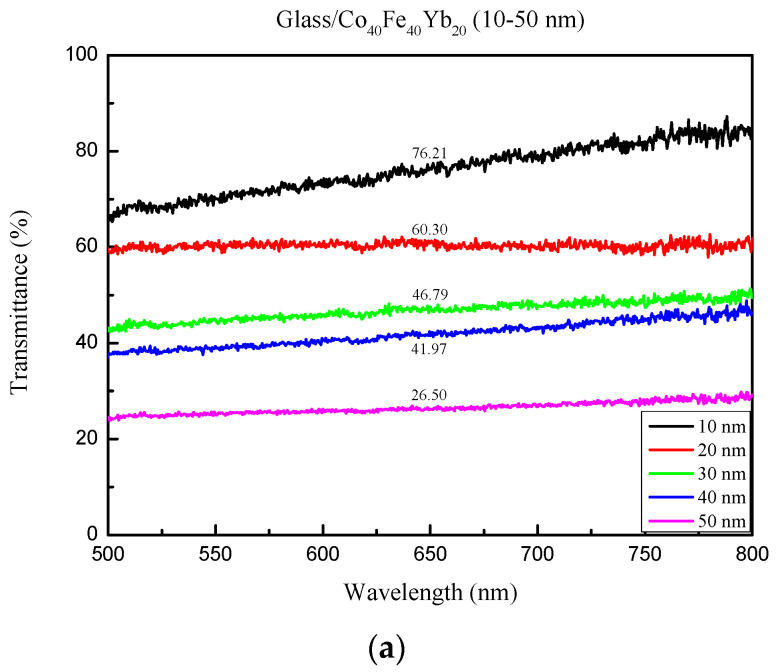

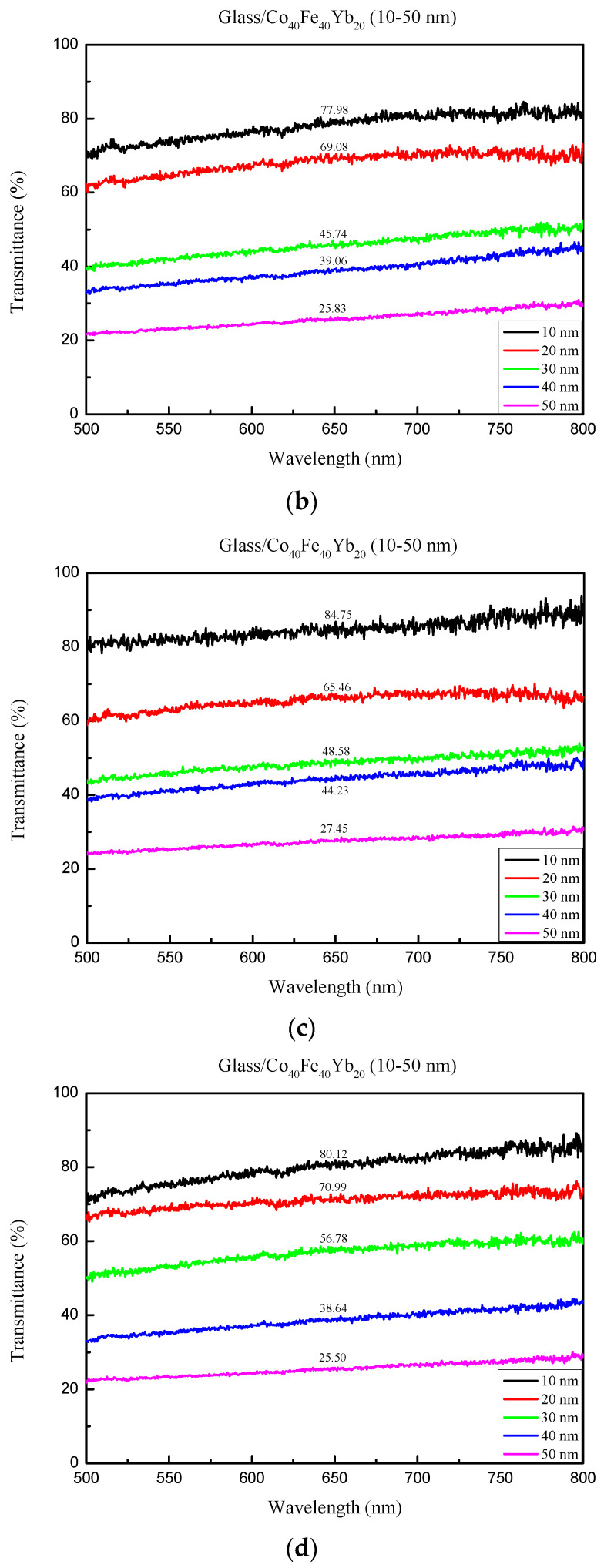
Transmittance of Co_40_Fe_40_Yb_20_ films. (**a**) RT, (**b**) following annealing at 100 °C, (**c**) following annealing at 200 °C, and (**d**) following annealing at 300 °C.

Figure 4 depicts the optical transmittance (%) spectra at visible wavelengths ranging from 500 nm to 800 nm. As shown in Figure 4a, as the thickness increased from 10 to 50 nm, the transmittance at RT decreased from 76.21% to 26.50%. Figure 4b shows that at a temperature of 100 °C, the transmittance decreased from 77.98% to 25.83%. Figure 4c shows that the transmittance decreased from 84.75% to 27.45% at the 200 °C annealing temperature, while Figure 4d shows that the transmittance decreased from 80.12% to 25.50% at the 300 °C annealing temperature. Only a minor change in transmittance was observed as the annealing temperature increased, and the trend was not immediately apparent. These findings imply that thinner Co_40_Fe_40_Yb_20_ films have a higher transmission rate because thicker films obstruct the incident signal [26,27,28]. The increased grain size with thickness and annealing treatment can be attributed to the improved crystallinity. Crystallinity increases due to the increased ability of add atoms to move to stable sites in the lattice. Thicker films are more homogeneous, resulting in fewer defects and localized states and an increase in the optical band gap [29].

### 3.4. Electrical Analysis

**Figure 5:**
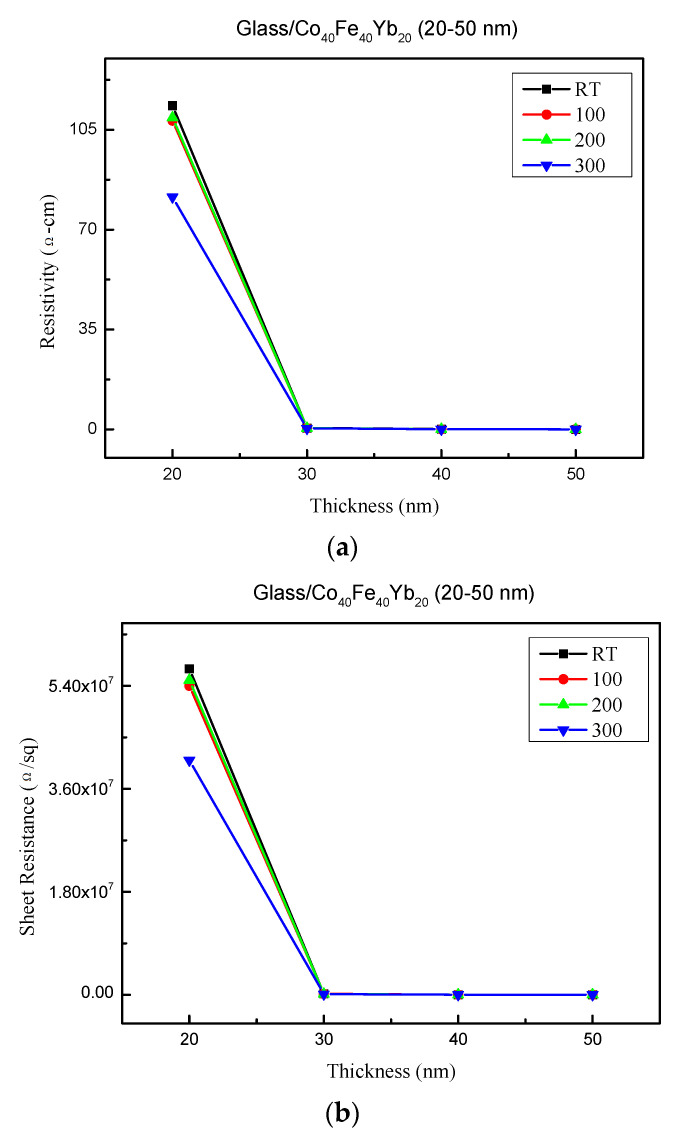
(**a**) Resistivity of Co_40_Fe_40_Yb_20_ thin films. (**b**) Sheet resistance of Co_40_Fe_40_Yb_20_ thin films.

The resistivity and sheet resistance are depicted in Figure 5a,b under all conditions. The resistivity at 10 nm was not tested because there was insufficient thickness to be examined. The sheet resistance and resistivity of Co_40_Fe_40_Yb_20_ films varied with thickness and annealing treatment. The conduction mechanism of CoFeYb film is thought to be related to the carrier present in the structure. The electrical properties of CoFeYb films are strongly influenced by their as-deposited and annealed environments. However, higher annealed temperatures and thicker thicknesses appear to result in better crystalline structure and lower carrier hindrance in the films, which is the dominant factor of conductivity and leads to lower resistivity. The results show that the sheet resistance and resistivity of CoFeYb films prepared using annealing treatments are lower than those obtained using unheated substrates [30]. In total, the resistivity varied between 0.02 and 113.47 Ω-cm, and the sheet resistance varied between 4.5 × 10^3^ and 5.7 × 10^7^ Ω. The resistivity and sheet resistance of as-deposited and thinner films are higher than those of annealed and thicker films because they have lower electron mobility through the film and more defects and impurities, which increase the presence of electron scattering [31,32].

### 3.5. Hardness Analysis

**Figure 6:**
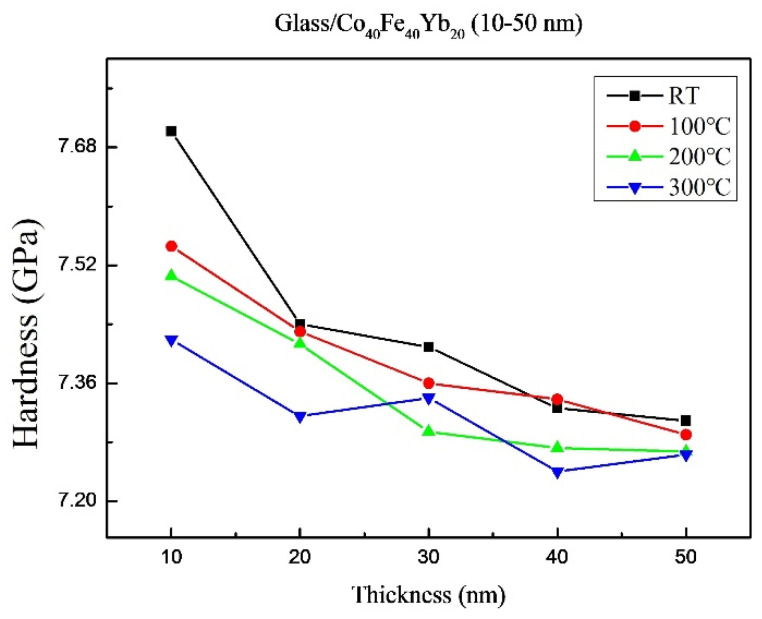
Average hardness of the Co_40_Fe_40_Yb_20_ thin films.

Figure 6 demonstrates that the films’ hardness ranged from 7.24 GPa to 7.70 GPa under all circumstances. The hardness shows a decreasing trend as thickness increases. As-deposited films have a harder surface than annealed ones. As the penetration depth increased, the hardness of the thin film with varying glass substrate thicknesses decreased; this could be a characteristic of the substrate when the penetration depth is sufficient [33,34]. The Pharr–Oliver method is commonly used to calculate hardness from loading and unloading curves, which demonstrate the combined hardness of the glass substrate and CoFeYb films [35]. Because of the thinness of the CoFeYb film, it is reasonable to assume that the substrate effect in nano-indentation measurement exists [36,37]. Furthermore, as the thickness increases, the hardness decreases, which is consistent with the Hall–Petch effect [38]. Because the harness value of annealing 300 °C thin film at a thickness of 30 nm is exhibiting abnormal behavior, it can be reasonably concluded that higher annealing treatment induces oxidation or impurity, resulting in dislocations that are difficult to move and increasing hardness [39]. 

### 3.6. Magnetic Analysis

**Figure 7:**
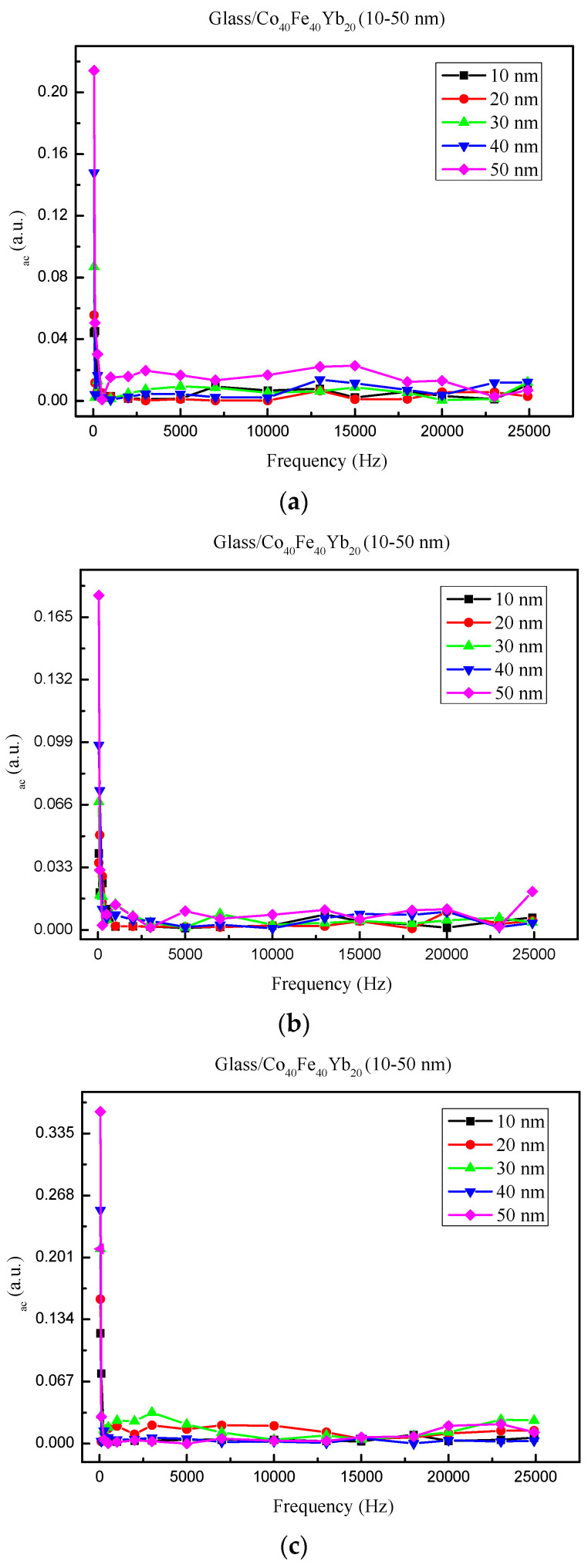

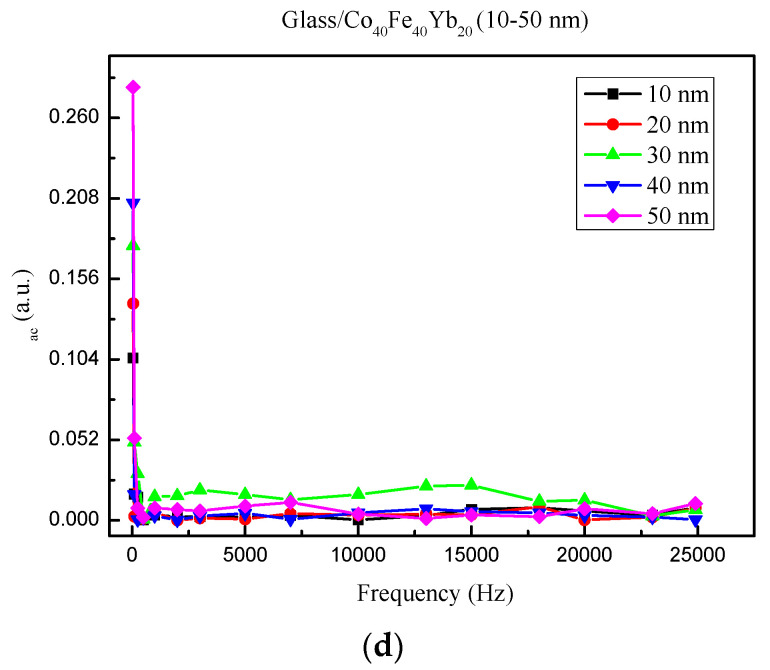
The relationship between the low-frequency alternate-current magnetic susceptibility (χ_ac_) and frequency. (**a**) as-deposited, (**b**) post-annealing at 100 °C, (**c**) post-annealing at 200 °C, (**d**) post-annealing at 300 °C.

Figure 7a–d depict the low-frequency alternative-current magnetic susceptibility (χ_ac_) under four conditions. The χ_ac_ decreased as the frequency increased in the 50–25,000 Hz range. As the film thickness increased from 10 to 50 nm, the corresponding χ_ac_ value increased. At high frequency, the χ_ac_ values of all films dropped dramatically. The maximum χ_ac_ value for as-deposited 50 nm was 0.214. The maximum χ_ac_ value for the 50 nm film was 0.176 after post-annealing at 100 °C, 0.358 after post-annealing at 200 °C, and 0.280 after post-annealing at 300 °C. 

**Figure 8:**
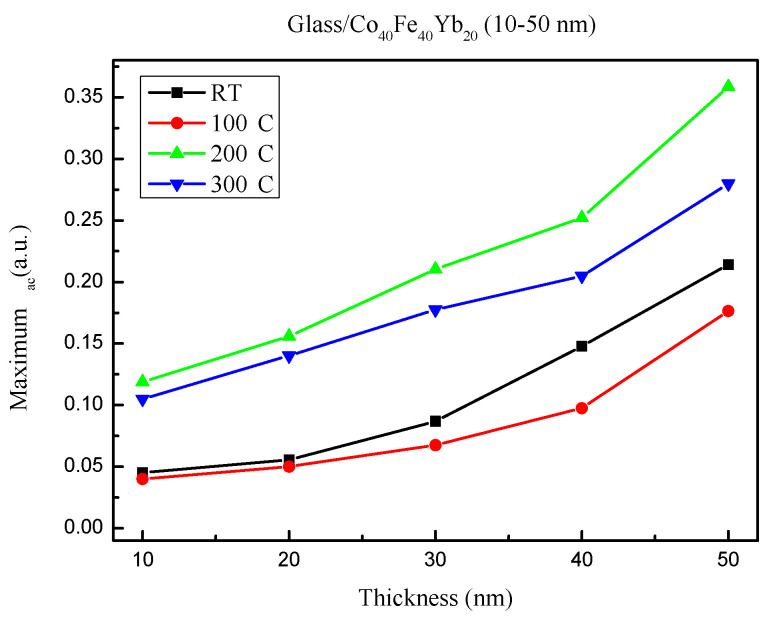
Maximum alternate-current magnetic susceptibility for Co_40_Fe_40_Yb_20_ thin films.

Figure 8 depicts the corresponding maximum χ_ac_ values under all conditions. Because of the thickness effect, the maximum χ_ac_ increased [40]. The annealing treatment produced the highest ac value of 0.358 at post-annealing 200 °C of the 50 nm film. Furthermore, due to thermal disturbance, the ac of post-annealing at 300 °C was lower than that at 200 °C.

The maximum χ_ac_ for the optimal resonance frequency (f_res_) under four different conditions is shown in Table 4. At the optimal resonance frequency, the maximum χ_ac_ exhibited the strongest spin sensitivity [41]. The f_res_ value was less than 100 Hz at each thickness. The optimal resonance frequency was determined to be less than 500 Hz, making it appropriate for use in low-frequency sensors, transformers, and magnetic components.

**Figure 9:**
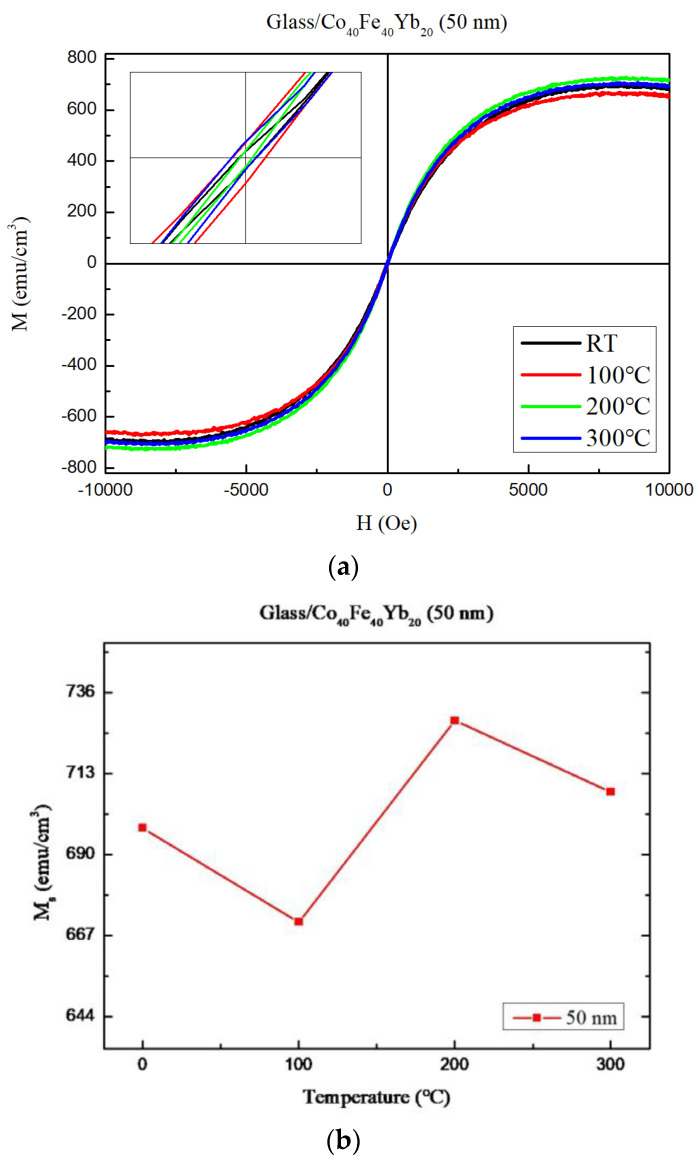
(**a**) In-plane magnetic hysteresis loop of Co_40_Fe_40_Yb_20_ thin films at 50 nm. (**b**) Saturation magnetization (M_S_) of Co_40_Fe_40_Yb_20_ at 50 nm.

The maximum χ_ac_ value of the film is 50 nm. As a result, the magnetic properties of a 50 nm film were investigated at various annealing temperatures. Figure 9a depicts the magnetic hysteresis loops of the 50 nm under four different conditions. With an external magnetic field (H_ext_) of 10 kOe in the plane, the saturated magnetic spin state was visible. The expanded figure shows low Hc, implying that the Co_40_Fe_40_Yb_20_ films have soft magnetization. The saturation magnetization (Ms) of the Co_40_Fe_40_Yb_20_ thin films is shown in Figure 9b under all conditions. The maximum value of Ms is comparable to the χ_ac_ result after post-annealing at 200 °C. Due to thermal disturbance, the Ms and χ_ac_ values of 50 nm films annealed at 300 °C were less than those at 200 °C. Ms decreased significantly after post-annealing at 100 °C, owing primarily to the temperature compensation effect [42].

In short, the underneath glass substrate has an effect on significant properties, which is worth discussing. The glass substrate itself is an amorphous structure, so even after heat treatment, the film is still an amorphous structure, resulting in no magnetocrystalline anisotropy and reduced magnetic property, surface energy decreases, transmittance increases, and grain refinement leads to an increase in hardness [43]. 

### 3.7. Challenges and Prospects

A new soft magnetic material is Co_40_Fe_40_Yb_20_ thin film. In the future, the question and challenge will be whether it can be used as a free or pinned layer in the MTJ structure. The key question is whether spin polarization can generate high TMR when applied to magnetic fields, as well as have high PMA and improve magnetic recording density.

## 4. Conclusions

Because of the addition of Yb and insufficient thermal driving force for grain growth, XRD results reveal that the structure is amorphous. A decrease in transmittance indicated that the thickness and interfacial effects were responsible for the transfer of photon signals through the material. The resistivity and sheet resistance significantly decreased as the thickness increased. As-deposited films have a harder surface than annealed ones. Hardness decreased as thickness increased, which is consistent with the Hall–Petch effect. At a post-annealing temperature of 200 °C, which is consistent with χ_ac_, the greatest Ms for a 50 nm was observed. The Ms and χ_ac_ values of the 50 nm film when annealed at 300 °C were lower than those at 200 °C, due to thermal disturbance. As a result, it was discovered that a 50 nm thick film and a 200 °C annealing temperature produced the highest ac and Ms and the lowest resistivity. The films were suitable for use in magnetic storage devices at this temperature because the Ms and χ_ac_ values were at their highest. 

## Figures and Tables

**Table 1:** Abbreviations and full names of proper nouns.

Abbreviation	Full Name
XRD	X-ray diffraction
χ_ac_	low-frequency alternative-current magnetic susceptibility
ƒ_res_	optimal resonance frequency
MS	saturation magnetization
MRAM	magnetic random-access memory
PMA	perpendicular magnetic anisotropy
Ku	magnetic anisotropy constant
CoFe	cobalt iron
Hc	coercivity
Tc	Curie temperature
H_k_	magnetic anisotropy field
RE	rare-earth
Yb	ytterbium
YAG	yttrium aluminum Garnet
MTJ	magnetic tunneling junction
TMR	tunneling magnetoresistance
B	boron
RT	room temperature
DC	direct current
Ar	argon
GIXRD	grazing incidence X-ray diffraction
θ	contact angle
DI	deionized
MTS	mechanical testing and simulation
CSM	continuous stiffness measurement
AGM	alternating gradient magnetometer
Hext	external magnetic field

**Table 2:** Significant properties for CoFeV, CoFeW, and CoFeYb materials.

Materials	Maximum χ_ac_ (a.u.)	Surface Energy (mJ/mm^2^)
Glass/Co_40_Fe_40_V_20_ [15,16] 10–100 nm at RT	0.02–0.04	27.8–45.4
Glass/Co_32_Fe_30_W_38_ [17] 10–50 nm at RT and annealed conditions	0.02–0.52	22.3–28.4
Glass/Co_40_Fe_40_Yb_20_ 10–50 nm at RT and annealed conditions (Current research)	0.04–0.35	28.6–34.5

**Table 3:** Comparing contact angle and surface energy for Co_40_Fe_40_Yb_20_ thin films from different fabrication processes.

Process	Thickness	Contact Angle with DI Water (θ)	Contact Angle with Glycerol (θ)	Surface Energy (mJ/mm^2^)
As-deposited	10 nm	76.5°	70.9°	29.47
	20 nm	74.4°	67.9°	30.37
	30 nm	74.6°	67.1°	30.86
	40 nm	69.9°	65.3°	33.02
	50 nm	68.8°	65.1°	33.72
Post-annealing 100 °C	10 nm	81.3°	71.4°	28.63
	20 nm	84.0°	72.5°	29.19
	30 nm	79.1°	71.6°	29.45
	40 nm	74.1°	72.0°	29.90
	50 nm	79.8°	69.1°	30.61
Post-annealing 200 °C	10 nm	82.5°	71.9°	28.82
	20 nm	86.4°	72.8°	31.46
	30 nm	71.8°	69.6°	31.28
	40 nm	70.9°	70.4°	32.48
	50 nm	74.8°	66.3°	31.52
Post-annealing 300 °C	10 nm	79.7°	71.8°	30.17
	20 nm	71.1°	68.3°	32.45
	30 nm	77.5°	67.7°	32.35
	40 nm	77.1°	64.7°	34.54
	50 nm	74.8°	63.7°	34.23

**Table 4:** The optimal resonance frequency for various thicknesses of films.

Thickness (nm)	As-Deposited Optimal Resonance Frequency (Hz)	Post-Annealing at 100 °C of Optimal Resonance Frequency (Hz)	Post-Annealing at 200 °C of Optimal Resonance Frequency (Hz)	Post-Annealing at 300 °C of Optimal Resonance Frequency (Hz)
10	100	50	50	50
20	50	100	50	50
30	50	50	50	50
40	50	50	50	50
50	50	50	50	50

## Data Availability

The data presented in this study are available on reasonable request from the corresponding author.
